# The Effect of Platelet-Rich Plasma on Pain, Function, and Quality of Life of Patients with Knee Osteoarthritis

**DOI:** 10.1155/2013/165967

**Published:** 2013-12-09

**Authors:** Seyed Ahmad Raeissadat, Seyed Mansoor Rayegani, Marzieh Babaee, Elham Ghorbani

**Affiliations:** ^1^Physical Medicine and Rehabilitation Department, Shahid Modarres Hospital, Shahid Beheshti University of Medical Sciences, Tehran, Iran; ^2^Physical Medicine and Rehabilitation Department, Shohada-e-Tajrish Hospital, Shahid Beheshti University of Medical Sciences, Tehran, Iran; ^3^Physical Medicine and Rehabilitation Department, Shahid Beheshti University of Medical Sciences, Tehran 1989934148, Iran

## Abstract

*Background*. New studies in the management of knee osteoarthritis have focused on modern therapeutic methods stimulating cartilage healing process. In the present study, we evaluated the effects of 2 courses of leucocyte-rich PRP (LR-PRP) injections on patients' QOL and functions and also the relationship between the PRP concentration and mentioned variables. *Material and Methods.* Sixty-five patients were evaluated. For each participant, WOMAC and the native (Farsi) edition of the SF-36 questionnaire were filled. Two courses of LR-PRP injections with 4-week interval were used. After 6 months, SF-36 and WOMAC questionnaires were filled again for each patient. *Results*. 60 patients were included in the final analysis. The mean platelet concentrations and white blood cell in PRP was 5-fold increase and 220 per microliter, respectively. The mean total WOMAC revealed significant change (*P* = 0.001). In SF-36, the mean changes of 2 major physical and mental domains were meaningful (*P* = 0.001). *Discussion*. In our study, 2 injections of PRP, with 4-week interval, improved the pain, stiffness, and functional capacity. Improvements in QOL (both PCS and MCS) were meaningful after injections. These changes were more significant in physical domains. PRP injection may be an alternative therapy in selective patients resistant to current nonsurgical treatments of knee osteoarthritis.

## 1. Introduction

Osteoarthritis, the most common articular disease in humankind, results from defects in articular cartilage and has significant effects on the quality of life (QOL) of patients, especially the elderly. For this reason, the effects of osteoarthritis and related therapeutical interventions on the QOL and patients' functions have been assessed in different studies [1].

There are different methods used for alleviating the symptoms of patients with knee osteoarthritis (OA), including various medications and supplements (NSAIDs, glucosamine, and chondroitin-sulfate), intra-articular injections (glucocorticoids, hyaluronic acid), physical agents (prescription of appropriate braces, shoes and insoles, exercise therapy, laser therapy, application of heat and cold modalities, etc.), and surgical interventions [2–4]. Although some of these treatments have had short- and mid-term effects on improving patients' functions and decreasing the level of disability, there still remain controversial results about their effects on decreasing the amount of articular damage and slowing the rate of disease progression. It seems that existing treatments cannot change the pathophysiology of the disease [5, 6].

Considering the aforementioned issues, new studies have focused on modern therapeutical methods stimulating cartilage healing process and improving its damage, including application of matrix metalloproteinase inhibitors, gene therapy, cytokinase inhibitors, stem cells, and growth factors [1]. Growth factor effects have been evaluated extensively both in vivo and in vitro [7–9]. Known platelet growth factors stimulate the healing process and lead to partial modification of the damaged tissue [10, 11]. Platelet-rich plasma (PRP), with higher platelet concentrations than the mean blood measures, is one of the sources for growth factors [12]. In most studies, the effective platelet concentrations have been considered to be between 3 and 7 times the normal average measures, depending on the kind of application (skin, hair, musculoskeletal, etc.). By activation of the platelets, different growth factors available in alpha and dense granules initiate the healing chain. This chain includes three steps of inflammation, proliferation, and remodeling [13]. Various results have been published about applications of PRP in different fields such as skin and hair, thoracic surgery, ENT, and orthopedics [14, 15]. PRP use has also been evaluated in musculoskeletal disorders such as muscular injuries, Achilles tendonitis, and tennis elbow with satisfactory results [16, 17]. Clinical PRP studies on arthrosis have recently been increasing. It has been considered in these studies that platelet growth factors could be effective in the cartilage healing process and chondrocyte stimulation and hopes have been made on the ability to influence the pathophysiology of OA [18–20].

There are extensive ongoing studies about the effects of PRP on knee OA, some of which were pilot studies [21, 22]. In these studies, patients' symptoms and their functions have been improved significantly after the usual 3 courses of injections with 2-3-week intervals [3, 4, 23]. To date, there is no consensus on the number of injections, the most effective platelet concentration, injection intervals, and the length of long-term PRP effects [10, 24]. In the present study, we evaluated the effects of 2 courses of PRP injections with 4 weeks interval, on patient's QOL and functions and also the relationship between the PRP concentration and mentioned variables.

## 2. Materials and Methods

In this clinical trial, patients with knee OA referring to the Physical Medicine and Rehabilitation Clinic in Shahid Modarres Academic Hospital in 2011 were evaluated. Inclusion criteria were arthralgia since the past 3 months with radiologic evidence of articular damage (grades 1–4 of Kellgren-Lawrence scale) [25] based on knee OA criteria of ACR [26, 27]. Exclusion criteria included age over 75 years, history of diabetes mellitus, immunosuppressive and collagen vascular disorders, history or presence of cancer or malignant disorders, any infection or active wound of the knee, recent history of severe trauma to the knee, autoimmune and platelet disorders, treatment with anticoagulant and antiplatelet medications 10 days before injection, use of NSAIDs 2 days before injection, history of knee articular injections of corticosteroids during previous 3 weeks or use of systemic corticosteroids 2 weeks before PRP injections, hemoglobin measures of less than 12 g/dL and platelet counts of less than 150,000 per microliter, history of vasovagal shock, pregnancy, or breastfeeding, and genu valgum/varum greater than 20 degrees.

After selecting patients, targets, and method of conduction and presenting scientific evidences, benefits and possible complications of participating in the study were described by a physiatrist verbally and written information about mentioned issues was also presented. After signing the consent form (approved by the Ethics Committee of Shahid Beheshti University of Medical Sciences), they were included in the study. Patients' personal information such as age, gender, height, weight, BMI (body mass index), educational level, physical activity, symptom duration, and the grade of OA (based on Kellgren-Lawrence grading scale in simple radiographs) were registered. Then, for each participant the native (Farsi) editions of the SF-36 (Short Form-36) questionnaire for assessment of QOL and WOMAC (Western Ontario and McMaster Universities Arthritis Index) questionnaire for evaluation of patients' functions were filled by a physical medicine and rehabilitation resident. The SF-36 questionnaire is one of the most common and comprehensive questionnaires used as a standard health outcome measurement tool on international level. It contains two major domains: physical health and mental health. Each domain has 4 subcategories consisting of physical functioning, physical role, bodily pain, and general health for the physical component summary or physical health category; social functioning, emotional role, mental health, and vitality in the mental component summary or mental health aspect. Higher scores in this questionnaire imply better patient status [28].

The WOMAC questionnaire is among the tools used for evaluation of patients' functions in rheumatic diseases especially knee OA. Three domains of stiffness, pain, and functional limitation are measured in this questionnaire. Higher scores in this regard imply worse patient status [29].

For the process of PRP preparation and injection, participants were referred to Shahid Modarres Hospital Laboratory. The PRP processing was done using the Rooyagen kit (made by Arya Mabna Tashkis Corporation, RN: 312569). For preparing 4–6 mL of PRP with concentration of 4–6 times the average normal values, 35–40 mL of blood was first collected from the patient's upper limb cubital vein using an 18 G needle. Then, 5 mL of ACD-A was added to the sample as an anticoagulant. One mL of the blood sample was sent for complete blood count. The rest of the sample passed two stages of centrifuge (first with 1600 rpm for 15 minutes for separation of erythrocytes and next with 2800 rpm for 7 minutes in order to concentrate platelets). The final product was 4–6 mL of PRP containing leukocytes. The PRP quantification and qualification procedure was performed using laboratory analyzer Sysmex KX 21 and swirling and if approved, the injection was proceeded. As it was stated in some resources that anesthetic agents not only could have toxic effects on chondrocytes but also by changing the pH of the environment could influence the activation of platelet, no local anesthetic agent was injected [7]. Instead, patients were given a single dose of acetaminophen-codeine 2 hours before the injection. It was also stated in some studies that a factor helpful for the activation of platelets is the contact with endogenous collagen [7]. We did not use exogenous factor for the process of activation but let the platelets be in contact directly with the joint collagen to become active. The skin of the injection site was prepped and draped and the liquid PRP was injected in a sterile condition using a 22 G needle through the classic approach for intra-articular injection (suprapatellar or medial). After 15–20 minutes of rest, patients were asked to actively flex and extend their knees so that the PRP could spread evenly across the joint space before changing into gel.

Then, participants were sent home with a written order concerning the following issues. They were recommended to have relative rest 24 to 48 hours after injection and limit weight bearing on the injected joint. Meanwhile for reducing pain and inflammation, they were instructed to use cold therapy three times a day each time for 10 minutes. In the case of pain onset, they had permission to use 500 mg of acetaminophen and if persistent, acetaminophen-codeine could be used PRN. However, they were strictly prohibited to take NSAIDs, aspirin, or any steroids. Generally, participants were recommended to have mild-to-moderate levels of activity and increase it as tolerated. They could resume their usual activities of daily living (ADL) one week after injection. Meantime they were instructed with exercise therapy and ADL modifications.

There is no consensus about standard regimen of PRP treatment in musculoskeletal disorders. In different study protocols, average series of injection is two to three at two- to six-week intervals [2, 23]. Because inflammatory process and patient's symptoms usually subside in 2 weeks [2], we chose 2 series of injection with 4-week interval in order to pass enough time to alleviate patient's symptoms. In our study, the second injection was performed 4 weeks after the first. All of the participants were visited serially 4, 8, and 24 weeks after treatment. Meanwhile they were evaluated for the amount of acetaminophen consumption, pain, joint swelling, and stiffness. After 6 months, SF-36 and WOMAC questionnaires were filled again. Participants were informed about the prescribed medications and the necessity for following the orders, avoiding medications influencing platelet activity, and having communication with the project executer in case of any problem.

Final data before and after the treatment were imported and analyzed by SPSS v. 16. Normality of the data described by mean and variance was evaluated using Shapiro-Wilk's test. For comparing variables with normal distribution, paired *t*-test, independent *t*-test, and ANOVAs test were used. To evaluate nonnormal variables, nonparametric tests of Wilcoxon signed rank, Mann-Whitney, and Kruskal Wallis were applied. Qualitative variables were expressed with frequency and percent. For evaluating the relationship between quantitative variables, correlation coefficients of Pearson and Spearman were used.

## 3. Results

Sixty-five patients were evaluated in this study. Five patients were excluded from the study, 3 due to concomitant use of NSAIDs, 1 due to failure to participate in follow-up program, and 1 due to lack of interest to continue with treatment. Finally, 60 patients were included in the final analysis including 52 women (93.3%) and 4 men (6.7%). The mean age of participants was 56.90 ± 8.8 years. The mean BMI was 28.46 ± 4.59 kg/m^2^. Demographic data of the patients are demonstrated in Table 1. Variables of age, physical function and pain domains of SF-36 before treatment and physical function, general health and energy domains of SF-36 after treatment, and WOMAC-related subcategory of functional capacity and total WOMAC had normal distribution.

PRP preparations in this study contained leukocytes (LR-PRP).  Table 2 demonstrates the mean platelet concentrations and white blood cell in PRP and the mean platelet concentrations at base (whole blood).

The most important patients' complaint was injection site pain. In some cases, pain lasted up to 10 minutes after injection, decreased gradually, and continued as a dull pain at the injection site. Some patients complained of transient knee stiffness and even local pelvic pain and feeling of swelling. Pain in most of them was improved by following the instructions and acetaminophen consumption. No significant complication was observed except for transient increase in local pain and swelling.

**Figure 1:**
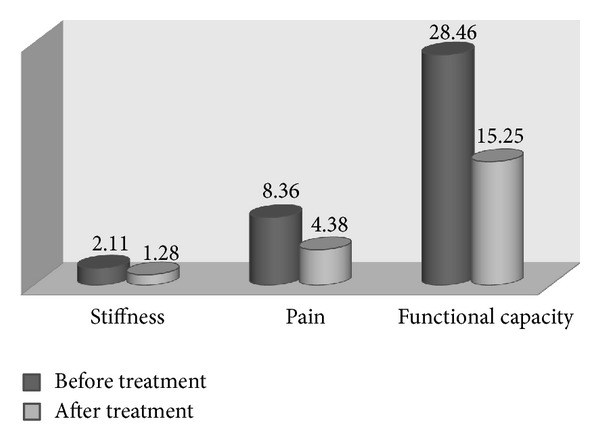
WOMAC index: pre- and posttreatment marks for pain, stiffness, and functional capacity. All results show significant improvement (*P* = 0.001).

The mean total WOMAC before treatment was 39.12 ± 16.25 and 21.05 ± 14.73 after treatment which experienced meaningful change (*P* = 0.001). The changes in all WOMAC subcategories were meaningful as demonstrated in Figure 1 (*P* = 0.001).

In SF-36, the mean changes of 2 major physical and mental domains were meaningful (*P* = 0.001). The mean change for mental component summary (MCS) before and after treatment was 51.11 ± 19.81 and 62.09 ± 22.09, respectively. The mean change for physical component summary (PCS) before and after treatment was 43.22 ± 16.36 and 62.02 ± 18.76, respectively.

**Figure 2:**
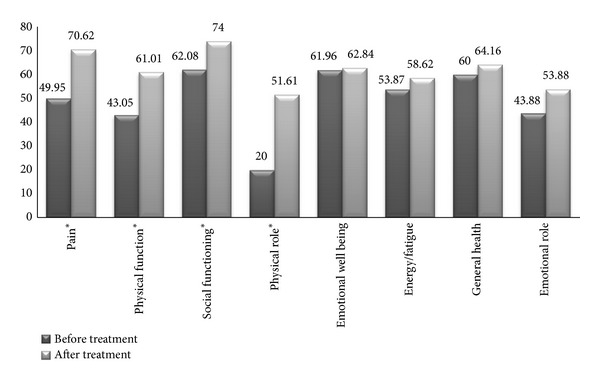
SF-36 test: pre- and posttreatment for the mental health domain and physical health domain. *These components were statistically significant.

All components of QOL improved in this evaluation among which the three variables of role limitation due to physical health, pain, and physical functioning changed meaningfully with *P* = 0.001. Social functioning also had meaningful improvement with *P* = 0.004 (Figure 2).

The amount of improvement in pain, stiffness, and functional capacity (evaluated by WOMAC questionnaire) had no meaningful relationship with any of the primary parameters (age, gender, educational level, symptom duration, physical activity level, and the grade of arthrosis) (*P* > 0.05).

The relationship between improved QOL (mental and physical health) and primary parameters (age, gender, educational level, the grade of arthrosis, physical activity level, and symptom duration) was evaluated and only the mean change of pain had relationship with age (*P* = 0.006, *R* = 0.353) while others lacked this relationship (*P* > 0.05).

The amount of improvement in joint pain, stiffness, and function and QOL had no relationship with patient's primary weight (*P* > 0.05). There was also no meaningful relationship between the mean concentration of platelets in PRP in first and second injections and mean improvement values of total WOMAC and SF-36 domains (*P* > 0.05).

## 4. Discussion

In our study, 2 injections of LR-PRP, with 4-week interval in between, improved the pain, stiffness, and functional capacity of patients with knee OA after 6 months. Improvements in QOL (both PCS and MCS) were meaningful after injections. These changes were more significant in physical domains (PCS) including role limitation due to physical health, pain, and physical functioning. Our results were similar to the study of Wang-Saegusa et al. [1]. They evaluated the effects of plasma-rich growth factor (PRGF) on function and QOL of patients with knee OA. In their study, the mean changes of WOMAC and related parameters and mean changes of physical parameters of SF-36 questionnaire were meaningful. In addition, the mean changes of mental parameters of SF-36 showed improvements; however, they were not meaningful. Sampson et al. studied the effects of PRP on primary and secondary OA in a pilot study. They also reported improvement in pain based on KOOS questionnaire and VAS evaluation [23].

Kon et al. evaluated the effects of PRP in short- (6 and 12 months) and long- (24 months) term in 2 separate studies. Similar results were obtained using IKDC questionnaire and VAS evaluation as assessment tools [3, 4].

In another study conducted by Kon et al., PRP, low- and high-molecular-weight hyaluronic acid were compared [30]. PRP was reported to be effective in improving patients' symptoms in addition to more pain reduction and longer effects comparing to hyaluronic acid. In contrast, Filardo et al. in a study comparing PRP and hyaluronic acid showed that although improvement in patients' symptoms after PRP injection lasted for one year; this improvement was not greater than hyaluronic acid in middle-aged patients with moderate signs. In that study, it was suggested that PRP should not be considered as the first-line treatment [31].

In the study of Patel et al. comparing the effects of single and two injections of PRP with normal saline injection (as control) in knee OA, a single injection of PRP was shown to be as effective as two injections and both were more effective than normal saline injection [32].

In our study, we analyzed the mentioned changes in WOMAC and SF-36 domains with demographic variables. None of them (age, gender, BMI, educational level, physical activity, symptom duration, and the grade of OA) has effect on the responses of the WOMAC and related parameters. A significant reverse relationship was found between patient's age and degree of pain reduction. Similarly, in the studies of Kon and Filardo, less reduction in IKDC scores was observed with advanced age. The observation of less responsiveness to PRP injection in advanced ages can be explained by the reduced number of available active and alive cells in order to react with growth factors. Furthermore, we evaluated the variable improvement results based on weight changes and found that there was no relationship between the amount of improvement in QOL, function, and weight changes. In contrast to our study, the amount of improvement was less with higher BMI [3, 4]. The difference between our study and the 2 mentioned studies may be related to patient selection (primary and/or secondary OA), primary BMI, and patients' age range.

In our study, although the clinical response rate was conversely related to severity of osteoarthritis, it was not statistically significant. This finding was not in agreement with other similar studies [3, 4]. It might be related to the different factors such as relatively small sample size of patients with grade 1 or 4 (the majority of patients had OA grade 2 or 3) and some severe cases with grade 3 or 4 were excluded from the study because of having exclusion criteria of genu valgum/varum more than 20 degrees and finally short-term followup.

In our study, the mean concentration of platelets in PRP was 3 to 7.8 times in the first injection and 2.4 to 8.6 times in the second injection. No relationship was found between the amount of improvement in pain, stiffness, functional capacity and QOL, and platelet concentration. Some studies mentioned that PRP could be effective in musculoskeletal diseases only in platelet concentrations of 4–6 times and concentrations of more than 8 times and less than 4 times had no such effect [6]. Even some believe that concentrations of more than 8 times can jeopardize the healing process and have inhibitory effects on cell proliferation process. However, as we know, a few articles have been published about the amount of PRP effectiveness in knee OA according to mean platelet concentration [10, 18, 24]. In addition, in our study PRP contained leukocytes with mean concentrations of 5–10 percent. To date, no human study has been published mentioning the mean leukocyte concentration in PRP. However, some studies have stated that leukocyte-containing PRP (LR PRP) could have some role in preventing injection site infection in addition to activating platelets and prolonging growth factor releasing time [33].

Generally, our study like others proposed the effectiveness of PRP in short term [20, 34, 35]. In our study, we tried first to evaluate the safety of our therapeutic protocol. Except for 10-minute pain at the site of injection and dull pain up to one week maximally, no other complication such as infection, atrophy, deep vein thrombosis, fever, hematoma, and tissue hypertrophy was observed (just like other studies). We also evaluated the therapeutic potential and availability of this method by assessing the primary findings and by conducting this study as a pilot study, we assessed the conditions for future studies.

Among the limitations of our study were the lack of control group and the relatively small sample size. The best PRP concentration, long-term effects, the number of injections and the intervals between, and the cost effectiveness of PRP are issues that necessitate more studies in comparison to control group and other current treatments. In addition, performing objective studies such as MRI and pathologic assessments would be useful in evaluation of PRP effectiveness in patients with OA.

## 5. Conclusion

Our study showed that intra-articular knee injection of PRP can decrease joint pain and stiffness and improve patients' QOL in short term. Therefore, PRP injection may be an alternative therapy in selective patients resistant to current nonsurgical treatments.

## Figures and Tables

**Table 1:** Demographic data.

Variables	Group characteristics	Number (percent)
Educational level	Below high-grade school diploma	27 (45%)
	High-grade school diploma and higher	33 (55%)

Dominant knee involvement	Right	23 (38.3%)
	Left	37 (61.7%)

Grade of tibiofemoral osteoarthritis	Grade 1	3 (5%)
	Grade 2	25 (41.7%)
	Grade 3	22 (36.7%)
	Grade 4	10 (16.7%)

Grade of patellofemoral osteoarthritis	Grade 1	6 (10%)
	Grade 2	21 (35%)
	Grade 3	19 (31.7%)
	Grade 4	12 (20%)

Regular physical activity (3 times a week, for at least 30 minutes every time)	Regularly active Not active	31 (51.7%) 29 (48.3%)

Symptom period	3–12 months More than 12 months	9 (15%) 51 (85%)

**Table 2:** PRP cytologic findings (mean ± SD).

Parameters injection	Platelets concentration in whole blood*	Platelets concentration in PRP*	Platelets concentration in whole blood/PRP^†^	WBC count in PRP*
First injection	224515.29 ± 70098.88	1285854.54 ± 494767.44	5.64 ± 1.16	220.68 ± 173.98
Second injection	224878.13 ± 79202.26	1304777.77 ± 374056.07	5.40 ± 1.54	685.71 ± 105.43

*Per microliter. ^†^Fold increase in platelet concentration.
